# A Theoretical Investigation of the Linear and Nonlinear Optical Responses of Scandium- and Yttrium-Doped (sub-nm) Ag and Au Clusters

**DOI:** 10.3390/ma19040678

**Published:** 2026-02-10

**Authors:** Munish Sharma, Mukesh Jakhar, Ravindra Pandey, Shashi P. Karna

**Affiliations:** 1 Department of Physics, School of Basic and Applied Sciences, Maharaja Agrasen University, Himachal Pradesh 174103, India; 2 Department of Physics, Michigan Technological University, Houghton, MI 49931, USA; mjakhar@mtu.edu; 3 Inter University Accelerator Center, New Delhi 174103, India; 4 DEVCOM Army Research Laboratory, Army Research Directorate, ATTN: CDD-RLA-A, Aberdeen Proving Ground, Aberdeen, MD 21005-5069, USA; shashi.p.karna.civ@army.mil

**Keywords:** doped Ag and Au clusters, NLO properties, TPA, OPA

## Abstract

We investigate the linear and nonlinear optical properties of (sub-nm) Ag_6_ and Au_6_ clusters doped with Sc and Y using time-dependent density functional theory. Both parent clusters have D_3h_ ground-state geometries but exhibit noticeably different electronic structures; scalar-relativistic corrections in Au_6_ induce significant s-d hybridization, resulting in larger HOMO-LUMO gaps and reduced one-photon absorption (OPA) cross-sections compared to Ag_6_. Two-photon absorption (TPA) peaks in the UV region show resonance enhancement via coupling with OPA-active states, with Ag_6_ having larger cross-sections than Au_6_. Doping with Sc and Y modifies the optical responses by breaking configurational symmetry and lifting HOMO degeneracies. ScAg_5_ and YAg_5_ energetically prefer planar configurations with higher dopant orbital contributions, while ScAu_5_ and YAu_5_ prefer non-planar configurations. This leads to blue-shifted, intensified OPA transitions and larger TPA cross-sections in doped clusters than in parent clusters. Doped Ag clusters exhibit a significantly stronger TPA response in the biologically relevant 1.8–2.0 eV (620–690 nm) spectral region for in vivo imaging. Furthermore, a higher degree of Sc(Y)-Au hybridization generates additional TPA pathways and also facilitates electronic transitions at 1064 nm, enhancing the first hyperpolarizability (β (−2ω; ω, ω)) for YAu_5_. Overall, the results show that these (sub-nm) Sc/Y-doped noble metal clusters are promising candidates for photonic and biomedical imaging applications.

## 1. Introduction

The (sub-nm) metallic clusters, including Ag_n_ and Au_n_, strongly interact with short laser pulses, producing distinctive optical responses based on their shapes and sizes [1,2,3,4,5,6,7,8,9,10,11,12,13,14]. For example, the Ag_n_ clusters (with *n* = 1–9) grown in various rare gas matrices have been reported to exhibit pronounced optical activity in the UV–visible region, significantly different from larger clusters, exhibiting discrete optical absorption features [4,5]. Experiments have also demonstrated that the (sub-nm) metallic clusters are efficient two-photon absorbers; a large two-photon absorption (TPA) cross-section, significantly higher than those for organic dyes, was reported for the thioguanine-conjugated gold cluster, Au_15_(SG)_13_ at 780 nm [9].

There has also been an intense interest in the use of rare-earth element doping to enhance the nonlinear optical (NLO) responses of host materials [15,16,17,18,19,20]. This may be due to the presence of multiple electronic states in rare-earth elements, which provide numerous pathways for NLO processes. These observations have motivated us to investigate (Sc, Y)-doped (sub-nm) Ag_n_ and Au_n_ clusters. It is expected that the doped systems will combine the unique properties of atomically precise metallic clusters with the optical characteristics of rare-earth dopants. The objective of this study is to investigate and understand the extent of dopant-induced modifications in the optical responses, specifically one-photon absorption (OPA), two-photon absorption (TPA), and dipole (hyper)polarizabilities of (sub-nm) Ag_n_ and Au_n_ clusters using time-dependent density functional theory (TD-DFT).

In this study, we consider Sc and Y atoms as dopants. These atoms possess a doublet spin state arising from their *d* orbital electron. The parent (host) clusters are the planar Ag_6_ and Au_6_ clusters, which have centrosymmetric ground state configurations that forbid second-order nonlinearity. Substitution of an Au/Ag atom in Au_6_/Ag_6_ by Sc/Y results in an overall singlet multiplicity (2S + 1) of the cluster. While the spin multiplicity of clusters has been found to correlate with (hyper) polarizabilities of rare-earth (RE) doped Au_5_-RE clusters [21], the role of relativistic effects on NLO properties has not yet been explored. It is important to note that in the case of Au, a relatively strong relativistic effect leads to the contraction of atomic shells compared to that in the case of Ag. Contraction of the Au-6s orbital leads to a relatively strong s-d hybridization, characterized by an enhanced covalent bonding nature in Au_6._ On the other hand, the Ag-5s electrons with a relatively large radial distribution dominate the bonding in Ag_6_. The effects of such differences in electronic structure and chemical bonding on the nonlinear optical properties of noble metal clusters have not been investigated to date. Another important molecular feature affecting the optical properties of molecule-like structures is the atomic geometry and symmetry. Previous studies have shown that ScAg_5_ prefers a planar configuration [20], while ScAu_5_ and YAu_5_ energetically prefer non-planar configurations [21,22,23,24]. Given such differences in electronic structure, bonding, and geometrical configurations, a natural question arises regarding their effects on the optical properties of Sc/Y-doped Ag/Au nanoclusters. To address this question, we investigated the linear and nonlinear optical properties, including OPA, TPA, and (hyper) polarizabilities, for the parent and doped clusters Au_6_, ScAu_5_, YAu_5_, Ag_6_, ScAg_5_, and YAg_5_ using the TDDFT method.

The structures, bonding, and optical spectra of the selected parent and doped clusters, except YAg_5_, have been the subject of previous experimental and theoretical studies [20,21,22,23,24]. While a comprehensive review of the literature is neither possible nor warranted here, a few relevant findings can be noted. An experimental study of the absorption spectra of Ag_n_ (4 ≤ 22) clusters isolated in an Ar matrix combined with a TDDFT study showed [3] that s-electrons were responsible for optical absorption in small clusters (*n* ≤ 8), while d-electrons dominantly participated in the observed spectra of larger (9 < *n* ≤ 22). A similar study on Ag_n_ (*n* = 1–9) clusters isolated in a solid neon matrix combined with DFT calculations [4] showed excellent agreement between experimental and predicted results for photon energies below 4.5 eV. Furthermore, this study showed that transitions in the energy range 3–4 eV were mainly due to s-electrons and were well separated from higher-energy transitions involving d electrons. Both these studies clearly showed the distinct characters of s- and d-electron excitations, with no mixing/hybridization between s- and d-electron orbitals, unlike that observed in Au. A more recent DFT study on Ag_n_ (*n* = 2–22) found, as in previous studies, that the Ag_6_ cluster is planar (D_3h_ symmetry) [25]. Additionally, the calculations showed that Ag_n_ clusters are planar structures for *n* = 3–6 and empty-cage structures for *n* = 7–18, with two minima in polarizability: one at *n* = 8 and the other at *n* = 18, with the value first increasing with *n* and then decreasing. The lowest polarizabilities at *n* = 8 and 18 were attributed to their closed-shell valence-electron configurations. A recent theoretical study of ScAg_n_ (*n* = 1–7) showed that the doped clusters for *n* = 4–7 adopt the planar configuration of their parent cluster. Furthermore, the doped ScAg_n_ clusters exhibited a delocalized bonding pattern between the dopant and the Ag host, which was termed σ aromaticity.

Several excellent reviews have been published on undoped and doped Au_n_ clusters [10,13,26,27,28,29,30]. A recent paper by Jakhar et al. calculated the structure, magnetic properties, and dipole polarizabilities of rare-earth (RE = Sc, Y, La-Lu) doped Au_5_RE clusters. As alluded to in the previous section, the calculations did not show a definite trend in the magnitude of hyper (polarizability) coefficients, α and β, due to dopants. However, it did show that the polarizability magnitude correlated with spin multiplicity of the cluster; i.e., larger polarizabilities were calculated for clusters with higher multiplicity.

## 2. Computational Method

The equilibrium geometry of the parent and doped clusters was calculated by Spin-polarized DFT calculations using the B3LYP functional form [31] as described in Jakhar et al. [21]. For Sc, Y, Ag, and Au, a scalar-relativistic effective core potential coupled with the Stuttgart/Dresden (SDD) basis set, was used. The SDD atomic basis sets were Sc: [Ar] 4s^2^ 3d^1^, Y: [Kr] 4d^1^ 5s^2^, Ag: [Kr] 4d^10^ 5s^1^, and Au: [Xe] 4f^14^ 5d^10^ 6s^1^, equivalent to double zeta functions [32,33,34]. The convergence criteria for the RMS density matrix and total energy were set at 10^−9^ and 10^−8^ Hartree, respectively. The configurations were optimized using the DALTON program (DALTON 2018.2 (2019)) until the force converged to 10^−4^ Hartree/Bohr [35]. Vibrational analysis was carried out for the parent and doped clusters, and only configurations with no imaginary frequencies were considered for further calculations of their optical properties.

For OPA calculations, the linear response function method [36] was used, as implemented in the DALTON program package [35,36]. In brief, the oscillator strength ($\delta_{OPA}$) is expressed in terms of the excitation energy ($\omega_{f}$) and transition dipole moment ($\mu^{0f}$) for a transition from |0〉→|f〉 state as $\delta_{OPA}=\frac{2\omega_{f}{\left|\mu^{0f}\right|}^{2}}{3}$, where $\mu^{0f}={\left(\sum_{\alpha }{\left|\langle 0|\mu_{\alpha }|f\rangle \right|}^{2}\right)}^{\frac{1}{2}}$ with $\mu_{\alpha }$ denoting the x, y, and z components of the transition dipole moment. Note that the scalar-relativistic SDD basis set provides reliable overall (5d → 6 s) excitation energies and trends in oscillator strengths. However, as SDD is a scalar-relativistic effective core potential (ECP), it averages relativistic effects and does not explicitly account for spin–orbit coupling. Consequently, the fine-structure splitting in the optical spectra is not captured in the SDD-based TD-DFT calculations performed in this study.

TPA, a third-order nonlinear process, was investigated using the residue of the quadratic response function (i.e., equivalent to a sum-over-state formulation with broadening), within the TD-DFT framework. Its implementation in the DALTON program package utilizes an atomic-orbital-based density matrix formulation [37,38,39,40]. The macroscopic TPA cross-section, $\sigma^{TPA}$, in atomic units, is calculated as

(1) $$\sigma^{TPA}=\frac{4\pi^{2}\alpha a_{0}^{5}\omega_{f}^{2}}{c\Gamma }\langle \delta^{TPA}\rangle$$

where $\delta^{TPA}$ is the TPA strength, *α* is the fine structure constant, *a*_0_ is the Bohr radius, c is the velocity of light in vacuum, Γ is a line-shape parameter (≈0.1 eV), and ω is the energy of the incoming photon.$\;\langle \delta^{TPA}\rangle \;$can be expressed as

(2) $$\langle \delta^{TPA}\rangle =\frac{1}{30}\sum_{ab=x,y,z}\left(S_{aa,bb}^{0f}\left(\omega \right)+S_{ab,ab}^{0f}\left(\omega \right)+S_{ab,ba}^{0f}\left(\omega \right)\right)$$

where the summation runs over the Cartesian components (*a* and *b*) of the symmetry-allowed elements of the dipole–dipole transition moment tensors (*S*) between the initial and final states.

It is known that the accuracy of the predicted two-photon absorption strengths from TD-DFT depends on the exchange–correlation functional since the excitation energy calculations require second-order derivatives of the functional with respect to variations of the density, and the TPA tensor requires evaluation of the corresponding third-order derivatives. The hybrid B3LYP exchange–correlation functional is a standard and effective choice for TD-DFT calculations in the present study, offering a good balance of accuracy and computational cost despite its known limitations for CT/Rydberg excitations [41]. On the other hand, the range-separated CAM-B3LYP functional does not improve TPA accuracy, as a benchmark study reported that it underestimates TPA values relative to those obtained with the coupled-cluster singles and doubles model (CC2) for a set of neutral fluorescent protein chromophores [42]. Furthermore, the TPA cross-section also depends on how accurately diffuse electron-density regions are described. Although SDD basis sets typically include fewer diffuse functions than explicitly augmented basis sets, such as aug-cc-pVnZ-PP, for heavier atoms, the SDD basis set, together with the B3LYP functional form, offers a balanced approach that requires modest computational resources.

In the present study, the OPA and TPA spectra were calculated for the low-energy, spin-allowed optical transitions involving 40 excited states, thereby accounting for nearly all low-energy optical transitions within these (sub-nm) clusters. Additional calculation with 100 excited states showed no discernible difference in the results for OPA and TPA, suggesting convergence at 40 excited states.

The linear polarizability (α) and first hyperpolarizability (β) were calculated using the TD-DFT implementation of the coupled perturbed Hartree–Fock approach [43,44,45]. The dipole moment, $\mu \;\mathrm{is}\;\mathrm{expressed}\;\mathrm{as}\;{\left(\mu_{x}^{2}+\mu_{y}^{2}+\mu_{z}^{2}\right)}^{\frac{1}{2}}$, the (average) linear polarizability, 〈α〉 is $\frac{1}{3}\;\left(\;\alpha_{xx}+\alpha_{yy}+\alpha_{zz}\right),$ and *β*_Total_ is $\;{\left(\beta_{x}^{2}+\beta_{y}^{2}+\beta_{z}^{2}\right)}^{\frac{1}{2}}$ with components in the x, y, and z directions, respectively.

## 3. Results and Discussions

### 3.1. Structural and Electronic Properties

#### 3.1.1. Parent Clusters

**Figure 1:**
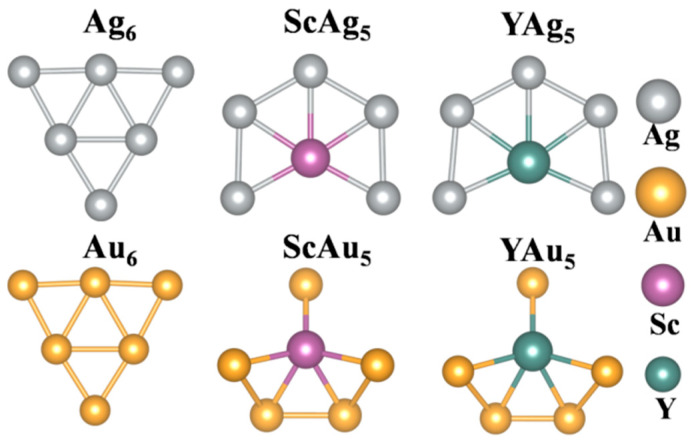
The equilibrium configurations of the parent and doped Ag and Au clusters. ScAg_5_/YAg_5_ prefers a planar configuration, while ScAu_5_/YAu_5_ prefers a non-planar equilibrium configuration.

Figure 1 displays the equilibrium configurations of the parent clusters. The calculated bond lengths, binding energy, and the energy gap between the highest occupied molecular orbital (HOMO) and the lowest unoccupied molecular orbital (LUMO) are listed in Table 1. The binding energy is with respect to the constituent atoms of a cluster.

The parent clusters have been the subject of several previous studies [8,21,25,46,47,48,49,50], which serve as good references for comparing the results of the present study. Ag_6_ and Au_6_ clusters prefer a planar, stacked-triangle configuration with D_3h_ symmetry. Furthermore, the calculated bond lengths (d_Au-Au_) agree well with the other theoretical results obtained, e.g., B3LYP/LANL2DZ (2.86 and 2.69 Å) [8], BLYP/DNP (2.78 Å and 2.63 Å) [46,47], B3LYP/SDD (2.87 and 2.69 Å) [21], and PW91/TZVPP (2.80 and 2.63 Å) [22] in the Au_6_ cluster.

**Figure 2:**
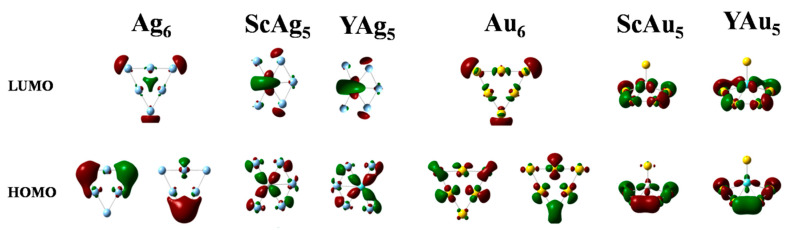
HOMO and LUMO orbitals of the parent and doped clusters (isosurface is 0.03 eV/Å^3^). The red (green) color represents the negative (positive) phase of the orbital.

The calculated (HOMO-LUMO) gap of Au_6_ is higher than that of Ag_6_. This results from relativistic effects in Au, which induce strong s-d hybridization in the molecular orbitals, leading to a large energy splitting between the bonding and antibonding orbitals. As displayed in Figure 2, HOMO of the planar parent clusters, Au_6_ and Ag_6_, appears to be primarily composed of doubly degenerate E′ orbitals, whereas LUMO is composed of a non-degenerate A_1_′ orbital. The HOMO-LUMO transition is thus symmetry allowed, albeit with a small transition dipole moment due to a weak spatial overlap between the primarily d-orbital HOMO and the more s-character LUMO states of the parent clusters (Table 2).

#### 3.1.2. Sc (Y)-Doped Ag and Au Clusters

Several isomeric configurations were generated from the parent cluster configurations by substituting Au or Ag with Sc or Y. We find that Sc (Y) doping yields an energetically preference for planar geometry in ScAg_5_ (YAg_5_) but a non-planar geometry in ScAu_5_ (YAu_5_). The planar configuration consists of a five-fold coordinated Sc (Y) atom, whereas the non-planar configuration consists of a quasi-planar pentagon in which Sc (Y) is bonded with a planar Au_4_ moiety. No imaginary frequencies are observed for the doped cluster configurations, suggesting that the configurations are stable after doping with Sc and Y.

The binding energy is calculated as $E_{b}=\;\frac{1}{6}\left[E_{doped\;cluster}^{Sc/Y}-\left(5\times E_{atom}^{Ag/Au}+\;E_{atom}^{dopant}\right)\right]$. A larger binding energy of the doped cluster suggests its increased stability, which may be due to the lowering of the configurational symmetry of the cluster after doping (Table 1). The predicted planar geometry of ScAg_5_ is consistent with the previously published literature [20]. Moreover, the calculated structural properties, including bond lengths for ScAu_5_ (YAu_5_) clusters, agree with those reported in previous DFT studies [21,22,23,24].

Figure 2 displays the dopant-induced modifications in the frontier molecular orbitals. Atomic Sc (Y) prefers a doublet spin state, but a charge transfer occurs from the dopant to the host atoms, yielding a singlet spin state for the doped clusters. This is consistent with the fact that Sc (Y) has a lower electronegativity than Ag (Au). Subsequently, the degeneracy in HOMO of the parent cluster is lifted, leading to a smaller electronic gap in the doped clusters (Table 1). As discussed later, the larger contributions of the dopant atomic levels in Sc(Y)Ag_5_ clusters compared to Sc(Y)Au_5_ clusters are reflected in their substantially different optical responses.

### 3.2. Optical Properties

#### One-Photon Absorption (OPA)

The calculated transition associated with the HOMO-LUMO states, and its oscillator strength, are listed in Table 2, while the OPA peak value of the transition associated with the largest δ_OPA_ is listed in Table 3.

In the parent clusters, the HOMO-LUMO transition follows the order indicated by the respective gaps, with *ω_H-L_* (Ag_6_) < *ω_H-L_* (Au_6_). For Ag_6_, the predicted optical transition is at 2.56 eV (484 nm) with a significantly low *δ_OPA_* of 0.04 a.u. For Au_6_, the transition is at 2.85 eV (435 nm), with a negligible *δ_OPA_* value of 0.005 a.u. As shown in Figure 2, the composition and symmetry of the HOMO and LUMO states of these parent clusters likely result in minimal spatial overlap, leading to a small transition dipole moment.

A strong optical response in the Ag_6_ cluster is predicted at 3.34 eV (375.7 nm) with *δ_OPA_* of 0.97 a.u (Table 3). The OPA peak is degenerate and closely aligned with the previously reported value of 3.31 eV [3], obtained from DFT (LANL2DZ/BP86) calculations. Experimentally, the dominant peak was reported at 3.45 eV (359.4 nm) for the Ag_6_ cluster embedded in an argon matrix [4]. In the Au_6_ cluster, the dominant non-degenerate transition with *δ_OPA_* of 0.19 a.u. occurs at 3.10 eV (400 nm) and corresponds to transitions between several frontier molecular orbitals (Table 3).

Sc- and Y-doping remove the degeneracy of the HOMOs in the parent clusters. The HOMO-LUMO transition in the ScAg_5_ (YAg_5_) clusters has a significantly low oscillator strength. In contrast, the doped Au clusters exhibit a higher value of the oscillator strength (*δ_OPA_* > 0.04) for the HOMO-LUMO transition, indicating a moderate one-photon activity (Table 2).

Table 3 shows that doping of the parent clusters results in a blue shift of the OPA peaks, which possess the largest oscillator strengths. This spectral shift can be attributed to significant rearrangements of the frontier molecular orbital energy levels, caused by the hybridization of Sc (Y) orbitals with those of Ag or Au. Overall, the dominant OPA transitions in the Ag clusters are predicted to be significantly more intense than those observed in the Au clusters. This difference is consistent with the greater degree of frontier orbital delocalization in Ag than in Au, where relativistic effects are dominant.

### 3.3. Two-Photon Absorption (TPA)

TPA within a specific cluster requires a long-lived virtual state between initial and final states and is accomplished by exciting a system with photons of longer wavelengths, thereby avoiding significantly stronger OPA. An energy range of 1–6 eV was selected for TPA calculations, following a theoretical study on thiolated Au_n_ clusters [37], which predicted a resonance-enhanced TPA peak at ~5 eV.

Table 4 displays the most prominent TPA transitions for the parent and doped clusters, along with their peak positions and absorption cross-sections. The results indicate that a resonance phenomenon involving the OPA states is responsible for the enhanced TPA, as the prominent peaks occur in the UV region, ranging from 4.65 to 5.45 eV (228–267 nm).

The reported TPA values (~10^8^ GM) represent idealized gas-phase upper bounds obtained from TD-DFT calculations with 0.1 eV Lorentzian broadening. The predicted qualitative TPA trends and rankings remain valid in the doped clusters, though they exceed the experimental values for ligated Au clusters [1,7] because ligand screening is absent and resonance conditions are ideal. The 0.1 eV broadening amplifies peak magnitudes per standard practice [39], whereas real solvent and broadening effects reduce values toward the experimental range.

Since TPA spectra calculated with monochromatic light depend on polarization (linear vs. circular), the polarization ratio, which characterizes how the TPA cross-section varies with polarization, is also listed in Table 4. A low value indicates a more isotropic excited state, while a high value indicates an anisotropic excited state. The results reveal contrasting excited-state characteristics for the parent clusters, Ag6 and Au6, with the former being highly anisotropic and the latter moderately so. This distinction arises from a less pronounced relativistic effect in Ag_6_ than in Au6, leading to more directional in Ag_6_.

A noticeable but small TPA cross section is predicted for Au_6_ (*σ^TPA^ ≈* 10^2^ GM), which can be attributed to relativistic effects that limit the available transition pathways among the quantized energy states of these (sub nm) gold clusters. Doping with Sc and Y shifts the TPA peaks with significantly larger TPA cross-sections. This is evident in the Au clusters and is likely due to the modified electronic states resulting from Sc (Y)-Au hybridization, which, in turn, increases the number of effective transition pathways for a TPA process.

**Figure 3:**
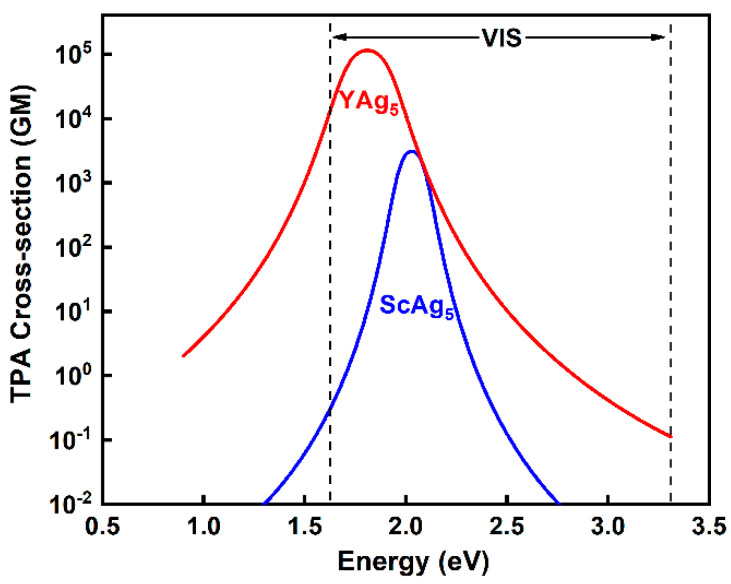
TPA peak positions and the corresponding cross-sections for ScAg_5_ and YAg_5_ in the spectral range of 600–700 nm (1.8–2.0 eV).

Next, we examine the TPA response in the near-infrared-to-visible spectral range of primary interest for photonic devices and biomedical imaging applications. The results reveal that Au_6_, ScAu_5_, and YAu_5_ have nearly zero TPA cross-sections for energies below 2.6 eV (470 nm), becoming non-zero (≈10 GM) afterward as we approach the corresponding OPA peaks. This is also the case with Ag_6_. However, Sc (Y) doping induces a significantly stronger TPA response (≈10^3^–10^5^ GM) in the energy range of 1.8–2.0 eV (600–700 nm), as shown in Figure 3. Interestingly, their OPA peaks are located in the low-energy range of 0.68–0.9 eV (1370–1820 nm), suggesting that the TPA response is associated with the OPA-enhanced resonance in this spectral range. The polarization ratio is 0.67 in both cases, indicating asymmetric electronic states with mixed character. Overall, doping modifies the cluster’s electronic structure, forming TPA-active states that effectively couple with incoming photons, thus demonstrating the effectiveness of Sc (Y) doping for enhancing TPA performance in Ag_6._

### 3.4. (Hyper) Polarizabilities

The polarizability (α) and the first hyperpolarizability (β) of both the parent and doped clusters were calculated using the TD-DFT method and are listed in Table 5. β (−ω; 0, ω), and β (−2ω; ω, ω) are the molecular hyperpolarizability coefficients corresponding to the electro-optic Pockels effect (EOPE), and second harmonic generation (SHG), respectively.

The parent Ag_6_ and Au_6_ clusters possess D_3h_ symmetry, resulting in zero dipole moment and first hyperpolarizability, (β). Doping with Sc and Y breaks the cluster symmetry, resulting in non-zero dipole moments and β (Table 5). The doped clusters show minimal changes in α due to Sc/Y doping but exhibit significant modulation in β, possibly via low-energy electronic transitions in the near-infrared region. Interestingly, while the static values of β for the ScAu_5_ and YAu_5_ are nearly the same, the frequency-dependent response at 1064 nm differs significantly. This is likely to be due to the alignment of the energy levels in YAu_5_, leading to a condition of near-resonance matching, thereby enhancing β at 1064 nm.

The calculated hyperpolarizability coefficient exhibits additional interesting features worth noting. We note that despite a planar minimum energy configuration and low dipole moment, (Sc/Y)Ag_5_, the α and β values are consistently larger than those of the nonplanar (Sc/Y)Au_5_, except for β (−2ω; ω, ω), which has a much higher value for YAu_5_. Between the Sc versus Y doping, the values for α and β in doped Ag clusters are consistently higher in the case of Y-doping than Sc, except for the β (−2ω; ω, ω), which is ~50% larger for in the case of ScAg_5_ compared to YAg_5_. This trend is reversed in the case of doped Au clusters, where Y-doping consistently gives larger values for μ, α, and β compared to Sc-doped clusters. These differences in the calculated dipole properties suggest an interplay among electronic structure, geometry, and relativistic effects in high-Z sub-nm clusters.

## 4. Summary

The linear and nonlinear responses of (sub-nm) Ag_6_ and Au_6_ clusters were investigated using time-dependent density functional theory. The calculated ground-state configurations of both parent clusters are predicted to be D_3h_, with HOMO-LUMO gaps exceeding 3 eV. While Ag_6_ experiences minimal relativistic effects, strong relativistic effects induce significant s-d hybridization in the frontier molecular states of Au_6_ due to the contraction of the s orbitals, which causes the s and d orbitals to approach each other in energy. This distinction between Ag_6_ and Au_6_ clusters is reflected in their optical responses, with a low OPA cross-section predicted for Au_6_. The prominent OPA peak with the largest oscillator strength is degenerate in Ag_6,_ but non-degenerate in Au_6_. Furthermore, the most prominent TPA peaks in the UV region exhibit resonance enhancement through coupling with OPA-active states, though Au_6_ shows relatively smaller TPA cross-sections than Ag_6_. Due to their D_3h_ symmetry, both clusters have zero dipole moment and static first hyperpolarizability (β).

Doping of Sc and Y in Ag_6_ and Au_6_ lowers the configurational symmetry, with ScAg_5_ (YAg_5_) being planar and ScAu_5_ (YAu_5_) being non-planar. It also lifts the degeneracy of the HOMO states and induces charge transfer between the dopant and host atoms. The composition of the HOMO (LUMO) states is slightly different, with contributions from the dopant atoms (Sc or Y) noticeably higher in the planar ScAg_5_ (YAg_5_) relative to the non-planar ScAu_5_ (YAu_5_) clusters. This, in turn, yields distinct optical responses in the doped clusters. The prominent OPA transitions with the largest oscillator strength, blue-shift upon doping, are more intense in Ag than in Au clusters. On the other hand, the prominent TPA peaks have significantly larger cross-sections compared to those in the parent clusters. Specifically, in the Au clusters, the modified electronic states resulting from a higher degree of Sc (Y)-Au hybridization appear to significantly increase the effective transition pathways for a TPA process. Within the spectral range of 620–690 nm, important for biomedical imaging applications, the doped Ag clusters exhibit much stronger TPA responses than the doped Au clusters. Furthermore, the cluster polarizability is not significantly modified by doping; however, doping facilitates electronic transitions at 1064 nm, thereby enhancing the first hyperpolarizability for YAu_5_. The (hyper) polarizabilities of the planar (Sc, Y) Ag_5_ have consistently higher magnitude compared to their nonplanar (Sc, Y) Au_5_ counterparts, except the β value corresponding to the SHG process for YAu_5_. Overall, the present study demonstrates that subtle differences in chemical bonding induced by the relativistic effects can result in distinct linear and nonlinear optical responses of Ag and Au clusters. These distinct optical responses can be leveraged to design and develop advanced devices for photonics and biomedical imaging applications. Although ligand and environmental effects were not explicitly modeled in this work, the substitution nature of Sc/Y doping suggests that the primary modifications to the electronic structure arise from the cluster core. Experimental stabilization with ligands, commonly used for sub-nanometer Ag and Au clusters [1,4,18], would mainly affect absolute magnitudes while preserving the relative trends predicted here.

## Figures and Tables

**Table 1:** The configurational symmetry, spin multiplicity ((2S + 1)), bond lengths (*d_Ag-Ag_*/*d_Au-Au_*; *d_X-Ag_*/*d_X-Au_*), binding energy (*E_b_*), and (HOMO-LUMO) gap (E_gap_) of the clusters.

Cluster		Symmetry	(2S + 1)	*d_Ag-Ag_* (*d_Au-Au_*) (Å)	*d_X-Ag_* (*d_X-Au_*) (Å)	E_b/atom_ (eV)	E_gap_ (eV)
Parent Cluster	Ag_6_	D_3h_	1	2.84, 2.72	-	−1.21	3.0
Doped Cluster	ScAg_5_	C_2v_	1	2.71	2.76	−1.31	1.9
Doped Cluster	YAg_5_	C_2v_	1	2.72	2.91	−1.41	1.8
Parent Cluster	Au_6_	D_3h_	1	2.87, 2.69	-	−1.51	3.4
Doped Cluster	ScAu_5_	C_S_	1	2.75	2.51	−1.96	2.2
Doped Cluster	YAu_5_	C_S_	1	2.75	2.70	−2.06	2.3

**Table 2:** Calculated HOMO-LUMO transition of the parent and doped clusters, including the peak energy (*ω_H-L_*) and oscillator strength (δ_OPA_).

HOMO-LUMO Transition			
	*ω_H-L_* (eV)	*λ_HL_* (nm)	*δ_OPA (H-L)_* (a.u.)
Ag_6_	2.56	484	0.038
ScAg_5_	0.68	1823	0.0003
YAg_5_	0.90	1378	0.0038
Au_6_	2.85	435	0.005
ScAu_5_	1.67	742	0.036
YAu_5_	1.95	636	0.075

**Table 3:** Calculated transitions with the largest oscillator strength of the parent and doped clusters.

	OPA Peak			States Involved in Transition
	*ω* (eV)	*λ* (nm)	*δ_OPA_* (a.u.)	
Ag_6_	3.34	376	0.97	(H) → (L + 1), (H − 1) → (L + 1), (H − 1) → (L + 2) (H − 1) → (L + 2)
ScAg_5_	3.65	340	0.55	(H − 2 → L + 5)
YAg_5_	4.14	300	0.56	(H − 3 → L + 2)
Au_6_	3.10	400	0.19	(H → L), (H → L + 1), (H − 1 → L + 2), (H − 1 → L + 1), (H − 1 → L + 2), (H → L + 3)
ScAu_5_	4.34	286	0.18	(H − 2 → L + 6)
YAu_5_	4.46	278	0.13	(H − 5 → L + 2), (H − 2 → L + 5)

**Table 4:** The most prominent TPA Peak position and corresponding cross-sections for the clusters. Cross-sections are computed using a Lorentzian line shape (Γ = 0.1 eV FWHM) at resonant conditions.

Cluster		TPA Peak Position (eV) (nm)		TPA Cross-Section (GM)	Polarization Ratio	Excited State
Parent	Ag_6_	5.13	242	6.72 × 10^5^	1.5	Highly Anisotropic
Doped	ScAg_5_	5.21	238	1.86 × 10^8^	0.67	Anisotropic
	YAg_5_	4.91	253	0.22 × 10^5^	0.67	Anisotropic
Parent	Au_6_	5.45	228	7.71 × 10^2^	0.21	Moderately Anisotropic
Doped	ScAu_5_	5.19	239	8.95 × 10^8^	0.72	Anisotropic
	YAu_5_	4.65	267	8.26 × 10^5^	0.67	Anisotropic

**Table 5:** Dipole moment (μ), (hyper) polarizabilities of the parent and doped clusters. α and β are linear polarizability and first hyperpolarizability, respectively. The frequency-dependent (hyper) polarizabilities are calculated at λ = 1064 nm.

Cluster	μ (Debye)	<α (0; 0)> (×10^−24^ esu)	<α (−ω; ω)> (×10^−24^ esu)	<β (0; 0, 0)> (×10^−30^ esu)	<β (−ω; 0, ω) (×10^−30^ esu)	<β (−2ω; ω, ω)> (×10^−30^ esu)
Ag_6_	0	41.1	44.7	0	0	0
ScAg_5_	0.4	41.9	49.8	16.1	24.4	107.2
YAg_5_	0.6	44.1	51.0	20.5	43.9	73.4
Au_6_	0	35.3	37.2	0	0	0
ScAu_5_	1.0	35.1	38.0	6.8	8.3	11.5
YAu_5_	1.4	36.9	39.7	8.9	34.4	265.0

## Data Availability

The original contributions presented in this study are included in the article. Further inquiries can be directed to the corresponding authors.
